# Hyperglycemia, a Neglected Factor during Cancer Progression

**DOI:** 10.1155/2014/461917

**Published:** 2014-04-17

**Authors:** Wanxing Duan, Xin Shen, Jianjun Lei, Qinhong Xu, Yongtian Yu, Rong Li, Erxi Wu, Qingyong Ma

**Affiliations:** ^1^Department of Hepatobiliary Surgery, First Affiliated Hospital, Medical College, Xi'an Jiaotong University, 277 West Yanta Road, Xi'an, Shaanxi 710061, China; ^2^Department of Anesthesiology, First Affiliated Hospital, Xi'an Jiaotong University, 277 West Yanta Road, Xi'an, Shaanxi 710061, China; ^3^Department of Pharmaceutical Sciences, North Dakota State University, Fargo, ND 58105, USA

## Abstract

Recent evidence from large cohort studies suggests that there exists a higher cancer incidence in people with type 2 diabetes (DM2). However, to date, the potential reasons for this association remain unclear. Hyperglycemia, the most important feature of diabetes, may be responsible for the excess glucose supply for these glucose-hungry cells, and it contributes to apoptosis resistance, oncogenesis, and tumor cell resistance to chemotherapy. Considering associations between diabetes and malignancies, the effect of hyperglycemia on cancer progression in cancer patients with abnormal blood glucose should not be neglected. In this paper, we describe the role that hyperglycemia plays in cancer progression and treatment and illustrate that hyperglycemia may contribute to a more malignant phenotype of cancer cells and lead to drug resistance. Therefore, controlling hyperglycemia may have important therapeutic implications in cancer patients.

## 1. Introduction

Hyperglycemia, or high blood glucose, is a condition in which an excessive amount of glucose circulates in the blood which develops when the body has too little insulin or when the body cannot use insulin properly. A number of medical conditions can cause hyperglycemia, including diabetes mellitus (DM) [1], obesity [2], pancreatitis [3], chronic stress [4], and cancer. Interestingly, the existing epidemiological evidence indicated that all of these hyperglycemia-related conditions are likely to be associated with tumorigenesis or tumor progression [5–7]. Nowadays, researchers mainly focus on the impacts of hyperglycemia on eyes, kidneys, nerves, and heart; little attention has been paid to the roles of hyperglycemia in cancer. Given the prevalence of hyperglycemia-related conditions existing in cancer patients, the relationship between hyperglycemia and cancer should arouse enough attention.

DM is the most common medical conditions responsible for hyperglycemia. In DM patients, blood glucose levels rise either because there is an insufficient amount of insulin in the body or the body cannot use insulin well. Diabetes mellitus has a current prevalence of 347 million people worldwide, and this number will continue to increase [8]. Epidemiologic evidence in the past suggested that people with diabetes are at significantly higher risk for many types of cancer [9]. It has been recognized that diabetes plays a crucial role in the development of solid organ malignancies including liver [10, 11], pancreatic [10, 12], colorectal [10, 13], breast [14, 15], endometrial [16–18], and bladder cancers [19, 20]. Among these cancers, liver cancer and pancreatic cancer (PC) show the strongest association with DM2. A recent meta-analysis of 23 articles demonstrated a 41% increase in cancer mortality related to endometrial, breast, and colorectal cancers in patients with preexisting diabetes compared with normoglycemic individuals [21]. Thus, many studies have provided consistent evidence on the association of diabetes with an increased risk of cancer. In contrast, diabetes occurs more frequently in patients with cancer than in the general population; therefore, new-onset diabetes may be an early indicator of subclinical cancer.

Following hyperglycemia, which was first reported in cancer patients in 1885, tumor tissues were found to sustain higher rates of glucose utilization than that in normal tissues by Warburg et al. in the 1920s [22]. Patients with various types of cancer have been examined in many clinical studies for proof of abnormalities in carbohydrate metabolism. The clinical evidence indicated a positive association between neoplasia and concomitant abnormalities in glucose metabolism. Moreover, several groups have described specific cellular mechanisms associated with glucose uptake in malignant tissues [23–25]. Most malignant tissues have increased fludeoxyglucose (^18^F) (^18^F-FDG) uptake associated with an increased rate of glycolysis and glucose transportation. The increase in ^18^F-FDG uptake noted in malignant tissue is related in a complex manner to the proliferative activity of malignant tissue and the number of viable tumor cells [26–28].

**Figure 1:**
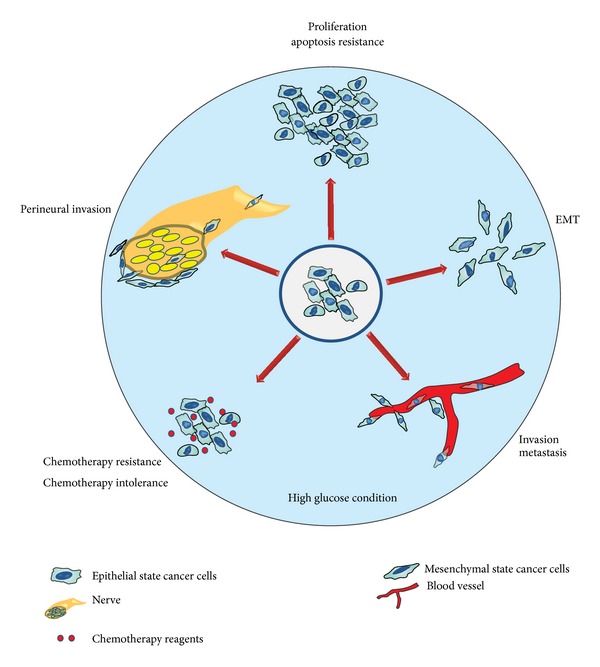
Hyperglycemia contributes to malignant cancer cell phenotypes. There is increasing evidence suggesting that there is a link between cancer and diabetes mellitus. Regardless of other shared metabolic factors, hyperglycemia, the most typical characteristic of diabetes, may be one reason to explain the prevalence of cancer incidence in patients with diabetes mellitus. Research shows that hyperglycemia may contribute to an enhanced proliferation ability, apoptosis inhibition, metastasis, perineural invasion, chemotherapy resistance, and chemotherapy intolerance.

Increasing evidence suggests a close association between diabetes and various malignancies; however, the potential biologic links between the two diseases are incompletely understood. Given that hyperglycemia is the most important biological feature of DM and cancer that are composed of glucose-hungry cells, it is not hard to imagine that hyperglycemia may play an important role during cancer progression in cancer patients with DM. Here, we review the available evidence of the relationship between hyperglycemia and different biological characteristics of cancer. It appears that hyperglycemia may contribute to a more malignant phenotype of cancer cells, including proliferation, apoptosis inhibition, metastasis, perineural invasion, chemotherapy resistance, and chemotherapy intolerance (Figure 1).

## 2. Hyperglycemia and Cancer Cell Proliferation

Glucose is specifically required to meet the metabolic demands of the fast proliferation cancer cells. It has been known that glucose is a primary driving force for the growth of tumor cells for more than two decades [29]. The promoting role of hyperglycemia on cancer proliferation is not hard to understand.

Hyperglycemia is often accompanied with hyperinsulinemia in people with DM2. Proliferation assays revealed that high levels of glucose (11 mM) and insulin (100 ng/mL) promoted the proliferation of the tumor cell lines HT29 (human colon carcinoma), SW480 (human colorectal carcinoma), MCF-7 (human breast adenocarcinoma), MDA MB468 (human breast adenocarcinoma), PC3 (human prostate cancer), and T24 (human bladder carcinoma) [30]. Furthermore, the addition of oral glucose, insulin injections, or both exhibited promoting effect on mammary tumor growth in rats [31]. Recent studies showed that insulin promote cancer progression by enhancing metabolic capacities of cancer cells [32]. Since high glucose and high insulin can induce cancer cell proliferation through different mechanisms, controlling blood glucose levels and insulin levels at the appropriate level would be beneficial in cancer patients bearing DM.

Under hyperglycemic conditions, studies have found not only an increased expression of the collagen receptor but also an integrin-linked kinase and other kinases regulating many cellular processes, including growth and proliferation [33]. There is some evidence that diabetes could promote PC cell proliferation. Chu et al. examined the records of resected PC patients and found that preexisting diabetes is associated with reduced survival in patients who underwent resection for PC. Additionally, PC with new-onset diabetes may exhibit increased tumor size and decreased postresection survival [34]. When hamster H2T pancreatic carcinoma cells were implanted into the cheek pouches of Syrian hamsters, the tumor size, weight, and total DNA content were significantly greater in animals with diabetes, demonstrating that diabetes appears to promote the growth of PC cells in the hamster [35].

**Figure 2:**
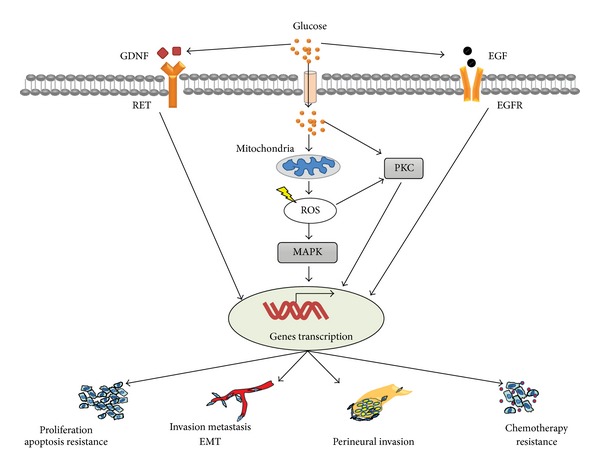
Mechanism of high glucose-induced cellular events in cancer cells. High glucose (hyperglycemia) generates cellular ROS mainly through mitochondrial metabolism; elevated ROS activate MAPK cascade, which cause cellular events by inducing related genes transcription. In addition, high glucose can induce activation of protein kinase C (PKC) through direct and indirect pathways. It is also speculated that high glucose may induce EGF transcription and EGFR transactivation, contributing to various biological behavior of cancer cells. High glucose-mediated GDNF upregulation may also involve in different cellular events through GDNF/RET cascade.

Increased production of reactive oxygen species (ROS) from mitochondria is the main cause of hyperglycemic complications (Figure 2). In diabetic individuals, hyperglycemia in susceptible cells results in the overproduction of superoxide by the mitochondrial electron transport chain [36]. Elevated levels of ROS can lead to cellular DNA mutations and may, therefore, play an important role in the initiation and progression of multistage carcinogenesis. More importantly, the generation of ROS was required for K-Ras-induced anchorage-independent growth through regulation of the ERK MAPK signaling pathway [37].

Hyperglycemia also specifically activates polyol metabolism with a subsequent decrease in Na^+^/K^+^-ATPase activity in pancreatic duct epithelial cells [38]. In addition, Tingstedt et al. found that the regenerating gene (REG) I-alpha protein was preferentially expressed in cancerous tissues and cells from PC patients with diabetes, and overexpression of this protein resulted in accelerated cell proliferation and consequently tumor growth in vitro and in vivo [39]. In addition, glucose concentration may be an important factor in breast cancer cell proliferation, and the prevalence of breast cancer is high in diabetic patients. The effects of glucose on breast cancer cell proliferation were evaluated by examining cell doubling time, DNA synthesis, the level of cell cycle related proteins, protein kinase C (PKC) isozyme expression, and the peroxisome proliferator activated receptor (PPAR) subtypes were determined following glucose exposure at normal (5.5 mM) and high (25 mM) concentrations in MCF-7 human breast cancer cells. In MCF-7 cells, high glucose stimulated cell proliferation as demonstrated by an increase in DNA synthesis and the expression of cdk2 and cyclin D1. The PKC-*α*, PPAR*γ*, and PPAR*α* protein levels were upregulated following high glucose treatment in drug-sensitive MCF-7 cells. These results suggested that hyperglycemia increases breast cancer cell proliferation through accelerated cell cycle progression with the upregulation of cdk2 and cyclin D1 [40]. Moreover, our group investigated the cell proliferation effects of glial cell line-derived neurotrophic factor (GDNF) and its tyrosine kinase receptor RET expression in BxPC-3 and MIA PaCa-2 cells with exposure to different glucose concentrations [41]. The proliferation of both BxPC-3 and MIA PaCa-2 cell lines was affected by glucose in a concentration-dependent manner. The definite expression of GDNF and RET was detected in both cell lines. The glucose concentration could alter the expression of GDNF and RET in a concentration-dependent manner correspondingly with an alteration in cell proliferation. The upregulation of GDNF and RET ligand-receptor interaction might participate in glucose-induced cancer progression (Figure 2). In addition, we demonstrated that the proliferative ability of BxPC-3 and Panc-1 cells was upregulated by high glucose in a concentration-dependent manner. Furthermore, the promotion effect of high glucose levels on EGF transcription and secretion but not its receptors in these PC cell lines was detected by using an EGF-neutralizing antibody and RT-PCR. In addition, EGFR transactivation is induced by high glucose levels in a concentration- and time-dependent manner in PC cells in the presence of an EGF-neutralizing antibody. These results suggest that high glucose levels promote PC cell proliferation via the induction of EGF expression and transactivation of EGFR [42].

## 3. Hyperglycemia and Cancer Cell Apoptosis

Apoptosis, a genetically regulated process that is essential to maintain individual homeostasis, is out of control in cancer. Resistance to apoptosis is one of the distinctive hallmarks of cancer cell [43]. High glucose condition is easy to induce apoptosis in normal cells [44, 45]. However, glucose metabolism protects cancer cells from cytochrome C-mediated apoptosis [46].

Recent in vitro studies suggest that an important mechanism for enhanced glucose metabolism in carcinoma cells involves the overexpression of transmembrane glucose transporters [47, 48]. Changes in glucose metabolism have also been found in many tumors, causing an increased production of lactate [49, 50]. Elevated lactate within cancer cells indicates a switch in glucose metabolism from the aerobic to anaerobic utilization of glucose, which was first described by Warburg (1956). Modern molecular biology has led to a renaissance in the Warburg effect [51, 52]. A permanent increase in anaerobic glucose utilization in primary tumors is a characteristic of more aggressive tumor cells [53]. Reduced mitochondrial respiration and an increased conversion of glucose into lactate combined with enhanced lactate secretion are associated with the acidification of a tumor and its environment [54]. This condition turns into an advantage for tumor cells with resistance to acidosis as realized by increased H^+^ transporter activity (e.g., Na^+^/H^+^ exchanger) [55]. However, in nonmalignant tissues, an acidic microenvironment is ordinarily toxic to mammalian cells, typically resulting in apoptosis through the activation of caspases [56].

Metformin, an oral antidiabetic drug in the biguanide class, is the first-line drug of choice for the treatment of DM2. The apoptosis-promoting effects of metformin on different cancers (e.g., ovarian cancer, breast cancer, and lung cancer) by increase of apoptotic genes have been demonstrated previously [57–59]. However, metformin-induced cancer cell apoptosis was prevented under high-glucose conditions in a carcinogen-induced rodent model of mammary tumorigenesis [60]. These data indicate that hyperglycemia can protect cancer cells from apoptosis process and thus failure to maintain glucose homeostasis may promote a more aggressive cancer phenotype.

## 4. Hyperglycemia and Cancer Cell Metastasis

Metastasis, which is considered a vital step in the progression of cancer, poses the largest problem for cancer treatment and is the main cause of cancer-associated deaths. Epidemiology studies showed that distant metastasis is responsible for nearly 90% of deaths from cancer [61–64].

Ever since metastasis has been investigated, models and concepts about how the metastatic disease process works have been suggested [65]. These include a “seed and soil” hypothesis in which a population of tumor cells are envisaged as seeds that require a suitable organ microenvironment, called the “soil,” to survive outside the primary tumor and grow as metastases [66, 67]. In the primary tumor site, reengineered cancer cells transform into an invasive phenotype to penetrate the tumor stroma and enter the blood circulation or lymphatic system via intravasation. In secondary lesions, a comfortable premetastatic niche must then be established for the traveling “seeds” forming macrometastases.

Recent studies revealed that hyperglycemia is associated with metastasis and might contribute to reengineer cancer cells in primary lesions. An epidemiology study demonstrated that, in cancer patients with DM2 or hyperglycemia, the proportion of tumor recurrence, metastasis, or fatal outcome is higher than in patients without metabolic disease [68]. Additionally, metformin, the most frequently used antidiabetic drug, inhibits cell migration and invasion by attenuating the cancer stem cell (CSC) function mediated by deregulating miRNAs including let-7a, let-7b, miR-26a, miR-101, miR-200b, and miR-200c, which are typically lost in PC [69]. In addition, metformin treatment also regulates the phenotype of CD44+/CD24− breast cancer stem cells by decreasing the expression of key EMT factors including the transcription factors ZEB, Twist, and Slug and the cytokine TGF-beta [70].

Vairaktaris et al. investigated the molecular basis of the association between oral cancer and diabetes (type I) in a rat model induced by a single intraperitoneal injection of streptozotocin dissolved in saline buffer [71]. This group observed that, although E26 transformation specific-1 (ets-1) expression was observed in diabetic and normal rats, its expression was higher in diabetic than normal rats in different cancer stages. It is widely recognized that ets-1 encodes a transcription factor that is involved in the transcriptional regulation of several genes implicated in tumor invasion and metastasis, such as collagenase I, stromelysin, and urokinase plasminogen activator [72–74]. Ets-1 has been implicated in human oral squamous cell carcinoma (OSCC), and ets-1 levels appear to correlate well with the grade of invasiveness and metastasis [75–77].

In recent years, the epithelial-mesenchymal transition (EMT) has received sufficient attention in metastasis. Cancer cells undergoing EMT obtain invasive properties and get into the surrounding tissue, leading to the creation of a suitable microenvironment for cancer proliferation and metastasis [78]. Accumulating data and studies have examined the relationship between EMT and hyperglycemia, mostly focusing on diabetic renal injury [79–81], diabetic vascular disease [82, 83], and peritoneal dialysis [84, 85]. Unfortunately, little attention has been focused on the role of hyperglycemia in inducing the cancer cell EMT phenotype. Our results demonstrated that high glucose could increase the production of ROS in the PC cell lines BxPC-3 and Panc-1, which further leads to cell motility and invasiveness [86]. We hypothesized that hyperglycemia facilitates PC metastasis by EMT induction and vascular destruction via oxidative stress [87].

## 5. Hyperglycemia and Perineural Invasion in Cancer

Perineural invasion (PNI) is defined as the presence of cancer cells within the epineural, perineural, and endoneurial spaces of the neuronal sheet and around the nerves [88, 89]. PNI is a distinct pathological entity that can be observed in the absence of lymphatic or vascular invasion, and it is associated with aggressive tumor behavior and worse clinical outcome [90]. Recent studies have demonstrated that hyperglycemia could facilitate PNI in several cancers, particularly pancreatic carcinoma [91, 92].

The mechanism of PNI in cancer is unclear. There are two prominent theories including the “path of low resistance.” There are three deficient sites around the perineurium: near the nerve ending, at the site invaded by the blood vessels present in nerves, and at the site invaded by the reticular fiber [93, 94]. Many previous studies presumed that tumor cells grow along the “path of low resistance,” and the path serves as a route for their distant migration [89]. Another possible explanation of PNI in PC is reciprocal signaling interactions. More recently, studies have demonstrated that PNI may involve reciprocal signaling interactions between tumor cells and nerves. These invading tumor cells may have acquired the ability to respond to proinvasive signals within the peripheral nerve milieu [89, 95]. The detection of increased neurotrophic factors such as nerve growth factor (NGF), glial cell line-derived neurotrophic factor (GDNF), brain-derived neurotrophic factor (BDNF), neural cell adhesion molecules (NCAMs), myelin-associated glycoprotein (MAG), and chemokines in intrapancreatic nerves and tumor cells and their receptors on tumor cells led to an increased attention to these molecules in recent years [96–98]. NGF and its receptor TrkA are the most widely observed among these factors. The receptor-ligand pair is overexpressed in PC cell lines and the perineurium of peripheral nerves. The binding of NGF to TrkA leads to the activation of the p44/42 MAPK signaling pathway, the promotion of cancer cell growth, increased invasiveness and metastasis, and eventually PNI mediation [99].

A recent study of 61 resected pancreatic tumors used histopathology to investigate many consecutive sections of tumor specimens, and the study reported an 86.9% (53/61) PNI rate in PC patients. Diabetic patients 93.75% (15/16) had a significantly higher frequency of PNI than nondiabetic patients 84.44% (38/45) [100]. A large retrospective study of 544 surgically resected pancreatic ductal adenocarcinoma patients observed similar results [101]. Diabetes or impaired glucose tolerance is often concurrently present in patients with PC and is associated with worse prognosis [34]. Nerve injury is a well-known complication of diabetes and is characterized by neuroinflammation [102]. Hyperglycemia in diabetes can cause up to a 4-fold increase in neuronal glucose levels. If persistent episodes of hyperglycemia exist, then intracellular glucose metabolism leads to neuronal damage [103, 104]. It is conceivable that, under hyperglycemic conditions, an increased level of oxidative stress and proinflammatory factors cause nerve damage and an inflammatory response [105], which simultaneously facilitates cancer cell proliferation, migration, and metastasis [106]. Li et al. revealed that nerve damage and regeneration simultaneously occur in the tumor microenvironment of PC patients with hyperglycemia; this simultaneous occurrence may aggravate the process of perineural invasion. The abnormal expression of NGF and p75 may also be involved in this process and subsequently lead to a lower rate of curative surgery [100]. In recent studies, researchers found that nerve invasion was dependent on GDNF secretion and mitogen-activated protein kinase activity. The GDNF coreceptors RET and GFR*α*1 were highly expressed in human pancreatic carcinomas by the same population of cells [96, 107–109]. Glucose concentrations could alter the expression of GDNF and RET in a concentration-dependent manner, and hyperglycemia could upregulate the interaction between GDNF and the RET ligand receptor [41].

In conclusion, hyperglycemia could promote PNI in several cancers, particularly pancreatic carcinoma. High glucose caused the demyelinization and axonal degeneration of nerves, which facilitate cancer cell invasion into nerves and enhanced interactions between nerve and cancer cells by increasing the expression of cytokines such as GDNF.

## 6. Hyperglycemia in Cancer Therapy

In addition to the effects of hyperglycemia on the biological behavior of tumor cells, the prevalence of transient hyperglycemia during induction chemotherapy has been observed, and existing evidence revealed another role of hyperglycemia in tumor treatment. There was evidence showed that hyperglycemia during chemotherapy for hematologic and solid tumors is correlated with increased toxicity [110]; thus, it appears that better glycemic control during chemotherapy could improve the toxicity and outcome of cancer patients. Moreover, hyperglycemia conferred resistance to chemotherapy for breast cancer but not nonmalignant cells, and this resistance was overcome by inhibiting fatty acid synthase (FAS) or ceramide production [111].

It has been known that DM patients were often accompanied by a disturbance in cellular innate immunity [112], and the impaired immune response may contribute to the ineffective chemotherapeutic treatment of cancer patients. For the past few years, epidemiological and laboratory evidence have shown that some antidiabetic pharmacotherapies exhibited outstanding effects for cancer prevention and treatment, such as metformin. Although some studies have revealed various molecular mechanisms for hypoglycemic agents and their anticancer effects, we should not neglect their glucose-lowering effects during cancer treatment because most malignancies constitute glucose-hungry cells. Collectively, controlling hyperglycemia may have important therapeutic implications for cancer patients. However, the role of hyperglycemia in cancer therapy and the exact mechanism remain unclear; thus, further studies are needed in this arena.

## 7. Conclusion and Future Directions

Increasing evidence has demonstrated a high incidence for various malignancies in patients with DM2. Although the shared associations between DM2 mellitus and cancer have been observed for a long time, the possible factors underlying cancer risk and mortality in this high-risk population remain uncertain. In this review, we discussed the effects of hyperglycemia, the key characteristic of diabetes mellitus, on various cancer biological behaviors and cancer treatment. In addition to providing rich nutrition for tumor growth directly, elevated glucose level could also induce the activation of some signaling pathways, all of which play important roles in cancer progression. Furthermore, hyperglycemiacanconfer resistance and intolerance to chemotherapy. Given the wide-ranging impact of hyperglycemia and the complexity of the microenvironment, the effect of hyperglycemia on the whole system and each component in a tumor microenvironment should not be neglected when exploring the relationship between cancer and diabetes mellitus. However, existing evidence indicates that hyperglycemia treatments may have important therapeutic implications in cancer patients.
